# Molting strategies of Arctic seals drive annual patterns in metabolism

**DOI:** 10.1093/conphys/coaa112

**Published:** 2021-01-05

**Authors:** Nicole M Thometz, Holly Hermann-Sorensen, Brandon Russell, David A S Rosen, Colleen Reichmuth

**Affiliations:** 1 Department of Biology, University of San Francisco, 2130 Fulton St, San Francisco, 94117 CA, USA; 2 Institute of Marine Sciences, University of California Santa Cruz, Long Marine Laboratory, 115 McAllister Way, Santa Cruz, 95060 CA, USA; 3 Alaska SeaLife Center, 301 Railway Ave, Seward, 99664 AK, USA; 4 Marine Mammal Research Unit, Institute for the Oceans and Fisheries, The University of British Columbia, 2202 Main Mall, Vancouver, BC V6T 1Z4, Canada

**Keywords:** Bearded seals, climate change, resting metabolic rate, ringed seals, sea ice loss, spotted seals

## Abstract

Arctic seals, including spotted (*Phoca largha*), ringed (*Pusa hispida*) and bearded (*Erignathus barbatus*) seals, are directly affected by sea ice loss. These species use sea ice as a haul-out substrate for various critical functions, including their annual molt. Continued environmental warming will inevitably alter the routine behavior and overall energy budgets of Arctic seals, but it is difficult to quantify these impacts as their metabolic requirements are not well known—due in part to the difficulty of studying wild individuals. Thus, data pertaining to species-specific energy demands are urgently needed to better understand the physiological consequences of rapid environmental change. We used open-flow respirometry over a four-year period to track fine-scale, longitudinal changes in the resting metabolic rate (RMR) of four spotted seals, three ringed seals and one bearded seal trained to participate in research. Simultaneously, we collected complementary physiological and environmental data. Species-specific metabolic demands followed expected patterns based on body size, with the largest species, the bearded seal, exhibiting the highest absolute RMR (0.48 ± 0.04 L O_2_ min^−1^) and the lowest mass-specific RMR (4.10 ± 0.47 ml O_2_ min^−1^ kg^−1^), followed by spotted (absolute: 0.33 ± 0.07 L O_2_ min^−1^; mass-specific: 6.13 ± 0.73 ml O_2_ min^−1^ kg^−1^) and ringed (absolute: 0.20 ± 0.04 L O_2_ min^−1^; mass-specific: 7.01 ± 1.38 ml O_2_ min^−1^ kg^−1^) seals. Further, we observed clear and consistent annual patterns in RMR that related to the distinct molting strategies of each species. For species that molted over relatively short intervals—spotted (33 ± 4 days) and ringed (28 ± 6 days) seals—metabolic demands increased markedly in association with molt. In contrast, the bearded seal exhibited a prolonged molting strategy (119 ± 2 days), which appeared to limit the overall cost of molting as indicated by a relatively stable annual RMR. These findings highlight energetic trade-offs associated with different molting strategies and provide quantitative data that can be used to assess species-specific vulnerabilities to changing conditions.

## Teaser

**Lay Summary**: We used fine-scale measurements of resting metabolism to define seasonal patterns of baseline energy demands for spotted, ringed, and bearded seals. These data reveal energetic consequences of different molting strategies and provide source data for population and bioenergetics models aimed at assessing the sensitivity of Arctic seals to environmental warming.

## Introduction

Rapid human-induced climate change is having disproportionate effects in the Arctic, where intense shifts in environmental conditions threaten the overall health and stability of Arctic and sub-Arctic ecosystems (Hinzman *et al.*, 2005; Post *et al.*, 2009; Serreze and Barry, 2011; Comiso and Hall, 2014). The present rate of change may be too rapid for long-lived and slowly reproducing species, such as marine mammals, to effectively adjust routine behavior or alter timing of life-history events. Associated threats to Arctic marine mammals include significant loss of sea ice habitats, changes in prey distribution and abundance, climate-induced stressors to body condition and health, and increased disturbance (Laidre *et al.*, 2008; Moore and Huntington, 2008). Rapid warming and dramatic declines in sea ice are of particular concern for ice-dependent Arctic seals, as loss of haul-out substrate could prevent individuals from successfully completing key life-history events and/or alter individual energy budgets. For many of these species, we lack appropriate data to make robust and meaningful predictions about the physiological impacts of environmental change. Specifically, directed studies examining energetic requirements and physiological constraints are urgently needed.

Resting metabolic rate (RMR) is a standard measure of individual energy expenditure, devoid of the influences of digestion, thermoregulation and reproduction. RMR is commonly used to compare energy needs across taxa (Kleiber, 1947; Thompson and Nicoll, 1986; Williams and Maresh, 2016), has been shown to scale to other measures of energy expenditure in marine mammals (Kooyman, 1989; Castellini *et al.*, 1992; Costa and Williams, 1999; Richmond *et al.*, 2006) and is a key component of many predictive modeling efforts (Winship *et al.*, 2002; Ophir *et al.*, 2010; Rechsteiner *et al.*, 2013; Villegas-Amtmann *et al.*, 2015; Costa *et al.*, 2016; Beltran *et al.*, 2017). Among phocids (i.e. true seals), RMR and overall energy budgets fluctuate annually in concert with environmental cues and internal biological cycles (Renouf and Noseworthy, 1990; Boily and Lavigne, 1997; Rosen and Renouf, 1998; Beck *et al.*, 2003; Sparling *et al.*, 2006). As a result, species-specific metabolic data—encompassing time-periods both inside and outside critical life-history events—are essential to more accurately forecast the consequences of sea ice loss for Arctic seals.

Although there are some studies of metabolism in Arctic seals (Gallivan and Ronald, 1981; Ashwell-Erickson *et al.*, 1986; Renouf and Gales, 1994; Ochoa-Acuña *et al.*, 2009), the majority of physiological data comes from field sampling of harvested animals that is often conducted in cooperation with native subsistence communities (Lenfant *et al.*, 1970; Ryg *et al.*, 1990a, 1990b; Lydersen *et al.*, 1992; Andersen *et al.*, 1999; Chabot and Stenson, 2002; Tryland *et al.*, 2006; Burns *et al.*, 2007; Quakenbush and Citta, 2008; Quakenbush *et al.*, 2009, 2011a, 2011b; Lestyk *et al.*, 2009; Ferreira *et al.*, 2011; Harwood *et al.*, 2012; Routti *et al.*, 2013). These sampling events generally take place in spring during annual breeding and molting periods when seals are most visible and accessible. Such efforts are useful for inter-annual comparisons of specific demographic groups but, due to the terminal nature of data collection, cannot provide fine-scale longitudinal data for individuals. Similarly, physiological data obtained during tagging expeditions are typically acquired once for each individual, as free-ranging Arctic seals are difficult to recapture for repeated measurements. Instrumentation secured to the pelage can be useful in collecting longitudinal behavioral data; however, the efficacy of such tags is reduced during the spring molt, as the probability of tag loss increases with molt progression. The overrepresentation of point-sampled data from individuals and data obtained from one period each year may bias our understanding of reference ranges for key parameters and limit our ability to understand the impact of specific life-history events on annual energetic profiles.

The molt is a significant physiological event that occurs following the breeding season each spring. During this time seals shed and re-grow their fur as well as several layers of epidermis. To facilitate this annual process, seals haul out for extended periods, increase blood flow to the skin and maintain elevated skin temperatures (Chang, 1926; Feltz and Fay, 1966; Boily, 1995). Hence, it has been proposed that the molt period should be characterized by increased metabolic rates; however, the cost of molt has been measured in a number of phocids with mixed results. Grey seals (*Halichoerus grypus*) (Boily, 1996; Boily and Lavigne, 1997), harp seals (*Pagophilus groenlandicus*) (Renouf and Gales, 1994; Hedd *et al.*, 1997; Chabot and Stenson, 2002), Hawaiian monk seals (*Neomonachus schauinslandi*) (Williams *et al.*, 2011) and southern elephant seals (*Mirounga leonia*) exhibit the predicted increase in metabolism coincident with molt. Northern elephant seals (*Mirounga angustirostris*) have been suggested to experience little to no energetic cost associated with molt (Worthy *et al.*, 1992). Alternatively, studies of spotted (*Phoca largha*) and harbor (*Phoca vitulina*) seals have documented decreases in metabolism during the molt (Ashwell-Erickson *et al.*, 1986; Rosen and Renouf, 1998). These conflicting findings suggest that the physiological consequences of molt are not fully resolved in phocids.

**Table 1:** Subject information for study animals, with age, mass and data collection dates presented as ranges across the sampling period. Location of study animals include the ASLC in Seward, AK, and the LML in Santa Cruz, CA

Species	Individual	Location	Sex	Age (yrs)	Mass (kg)	Data collection
*Phoca largha*	Amak	ASLC	M	5.8–9.2	49.2–85.0	Feb 2016—Jun 2019
	Tunu	ASLC	M	5.8–9.2	59.5–85.5	Feb 2016—Jun 2019
	Sura	ASLC	F	2.8–5.2	36.0–59.0	Feb 2017—Jun 2019
	Kunik	ASLC	M	1.8–4.1	37.0–59.0	Feb 2017—Jun 2019
*Pusa hispida*	Nayak	LML	F	5.5–8.1	24.9–32.5	Nov 2016—Jun 2019
	Pimniq	ASLC	M	3.5–5.2	25.3–31.7	Oct 2017—Jun 2019
	Dutch	ASLC	F	2.8–3.2	27.3–28.6	Jan 2019—May 2019
*Erignathus barbatus*	Noatak	LML	M	2.0–4.3	102.4–144.6	Mar 2017—Jun 2019

Given that the timing of molt is typically entrained to specific environmental cues like photoperiod (Ling, 1984; Mo *et al.*, 2000), and has evolved to coincide with particular sea ice conditions for Arctic seals, the ongoing and rapid loss of adequate haul-out substrate may have significant energetic implications for ice seals during this time. If seals must increase time in cold polar water during the molt, lowered skin temperatures could inhibit hair growth, resulting in prolonged, disrupted, or failed molt cycles (Boily, 1995). More time in water during molt may also lead to increased heat loss and an overall increase in the metabolic cost of molting. Alternately, seals may modify their behavior if suitable sea ice becomes unavailable in preferred areas, choosing instead to molt on substrate farther away from foraging grounds (Von Duyke *et al.*, 2020) and/or molt on land, which could raise both daily energy budgets (Hamilton *et al.*, 2015) and predation risk (Smith, 1980). Such behavioral changes will likely influence the degree to which Arctic seals can maintain energy balance.

In this study we use a comparative framework to examine annual patterns in metabolic requirements for three Arctic phocids that differ greatly in their use of and dependence on sea ice. Spotted seals are an ice-associated species found in both sub-Arctic and Arctic regions that utilize seasonal pack ice edges for pupping and molting, but may also use coastal haul-outs during the summer and fall in some locations (Burns, 1970; Lowry *et al.*, 1998). Ringed (*Pusa hispida*) and bearded (*Erignathus barbatus*) seals have circumpolar Arctic distributions and are strongly ice-dependent. Ringed seals maintain breathing holes and excavate subnivean lairs in order to remain in areas of extensive fast ice for much of the year, only hauling out on exposed sea ice for extended periods during the spring molt (Burns, 1970; Smith and Stirling, 1975). In contrast, bearded seals use broken and moving pack ice, particularly in regions over shallow foraging grounds, as a platform for pupping and molting (Burns, 1970; Breed *et al.*, 2018; Cameron *et al.*, 2018). Sea ice loss will impact the ability of all three species to successfully carry out important life-history events and alter individual energy budgets (Laidre *et al.*, 2015; Hamilton *et al.*, 2016); however, the extent and severity of effects will depend on the physiology, life-history characteristics and behavioral flexibility of each species.

Due to the remote habitats and cryptic behavior of these species, the only way to obtain year-round metabolic data is through the study of captive individuals. This approach enables the examination of fine-scale changes in parameters over time, under controlled conditions. Working with four spotted seals, three ringed seals and one bearded seal trained to participate in physiological sampling, we document fine-scale changes in the RMR of individuals over a four year-period, with particular focus on using within-subject repeated measures to determine the metabolic implications of molt. Here, we provide measures of RMR for each species, describe seasonal changes, examine potential drivers of observed patterns and discuss the relevance of our findings for each species in light of rapid sea ice loss and ongoing climate change.

## Methods

### Animals

Eight ice seals participated in this multi-year study at two facilities (Table 1). The spotted and ringed seals stranded at young ages in the wild and were brought to the Alaska SeaLife Center (ASLC) for rehabilitation. Individual seals spent an average of two to three months in rehabilitation and were deemed healthy by veterinary staff prior to entry into this study. The bearded seal was collected in the wild, brought to ASLC for one and a half months and then moved to Long Marine Laboratory (LML) at six months of age for participation in research. One ringed seal and one bearded seal were housed at LML, Santa Cruz, CA (36.9497° N, 122.0656° W). Four spotted seals and two ringed seals were housed at ASLC, Seward, AK (60.0999° N, 149.4410° W). Study animals included both males and females, as well as sub-adult and adult individuals. The seals were trained to voluntarily participate in data collection and routine husbandry care using positive reinforcement methods. Seals at both facilities were housed in natural seawater pools with water temperatures that ranged seasonally with local environmental conditions (LML T_w_ = 8.6 °C—19.3 °C; ASLC T_w_ = 4.0 °C—11.5 °C) and had access to haul-out areas at all times of day and during all seasons. TidBit v2 temperature data loggers (Onset Computer Corporation, Bourne, MA, USA) were used to measure and record ambient air and water temperatures at each facility at one-hour intervals. Mean daily air and water temperatures were determined by averaging hourly measurements for each 24-hour period. Seals were kept outdoors under local photoperiod at each facility.

Seals were fed a daily diet composed of several prey types that included relatively high-fat clupeid fish (e.g. herring—*Clupea* spp.) and relatively high-protein osmerid fish (e.g. capelin—*Mallotus villosus*), supplemented with cephalopod or bivalve mollusks. Vita-Zu marine mammal tablets (Mazuri, PMI Nutrition International LLC) were included in the diet to ensure proper nutrition. Metrics of food motivation and appetite were scored daily by experienced staff and used to determine optimal diet each day, which allowed caloric intake and body mass to vary seasonally in a natural manner. A subsample of each prey batch was analyzed for proximate and energetic analyses (Michelson Labs Inc., Commerce, CA) and daily food consumption was recorded in both mass (kg) and energy (kcal). Animals were weighed weekly to the nearest tenth of a kilogram using a calibrated platform scale.

Research was conducted under United States National Marine Fisheries Permit 18 902 issued to C. Reichmuth with authorization from the Ice Seal Committee. Institutional Animal Care and Use Committees at the University of California Santa Cruz and Alaska SeaLife Center approved research protocols and provided oversight of animal welfare.

### Metabolic measurements

In-water, RMRs were determined for all ice seals using an open-flow respirometry system designed for marine mammals. Individuals were trained to remain stationary beneath a custom-built metabolic dome attached to a PVC piping frame that floated at the top of the water surface (101 cm long x 90 cm wide x 46 cm high). Animals were able to move into and out of the dome freely, but were trained to voluntarily station and rest beneath the dome for extended intervals. Sessions were conducted in the morning following an overnight fast to ensure that seals were postprandial. To establish consistent and relaxed cooperative behavior, seals rehearsed this routine for 4–8 min each morning and were rewarded with 30 to 40% of their scheduled diet. Data collection trials were attempted weekly with average duration ranging between 10–13 min depending on the pre- and during-trial behavior of each animal on a given day. RMR measurements were considered useable only when animals were at continuous rest beneath the dome for a minimum of 5 min, and the metabolic trace contained an interval of resting equilibrium that exceeded 4 min.

Rate of oxygen consumption ($\dot{\textrm{V}}$O_2_) during metabolic trials was determined by pulling ambient air through a metabolic dome at known rates between 150–200 L/min by a mass flow controller (Sable Systems International, North Las Vegas, NV, USA). Exact flow rates were dependent on individual mass and set to ensure that the oxygen content within the dome was maintained above 20.10% at all times. Subsamples of dome exhaust were dried (Drierite, W. A. Hammond Drierite, Xenia, OH, USA), scrubbed of carbon dioxide (Sodasorb, Smiths Medical Inc., Minneapolis, MN) and dried again, before entering an oxygen analyzer (Sable Systems International, North Las Vegas, NV, USA). Oxygen content of dome exhaust was recorded every 1.0 s on a laptop computer using EXPEDATA software (Sable Systems International, North Las Vegas, NV, USA). Ambient air baselines were collected before and after animals were under the dome to account for system drift and for use in $\dot{\textrm{V}}$O_2_ calculations. Temperature and relative humidity of chamber air was determined during trials using a handheld temperature and humidity sensor (Vaisala HM40, Vantaa, Finland). Flow rates were corrected to standard temperature and pressure dry (STPD) and $\dot{\textrm{V}}$O_2_ determined using EXPEDATA software (Sable Systems International, North Las Vegas, NV, USA) and standard equations (Withers, 1977). Before each trial the oxygen analysis system was calibrated with dry, ambient air. Metabolic systems at both facilities were routinely checked for leaks and accuracy using 100% nitrogen gas (Fedak et al 1981; Davis *et al.* 1985).

### Molt status

We documented the timing, progression and overall duration of the visible molt for each animal annually. Data on molt were compiled from detailed photographic records, molt monitoring data sheets and husbandry records. The start date of each molt was defined as the first documentation of loose hair and/or active hair loss. The end of each molt was defined by the complete loss of the old coat and complete re-growth of a new coat. The 50% molt date was the day at which half of the new coat was determined to have grown in. For statistical analyses the molt status of an individual for a given metabolic data collection session was categorized in one of four ways: not molting, 1 month pre-molt, actively molting or 1 month post-molt.

### Statistical analyses

We did not assume each species would respond similarly to changes in fixed parameters and therefore, evaluated each species independently.

To examine potential drivers of metabolism in ringed and spotted seals we used linear mixed-effects (LME) models. We included individual as a random effect within each LME model for each species, which allowed us to account for repeated measures and avoid issues associated with pseudo-replication (Harrison *et al.*, 2018). Absolute $\dot{\textrm{V}}$O_2_ data (rather than mass-specific) were used in statistical analyses to avoid any *a priori* bias based on scaling (Nagy, 1987; Glazier, 2005). We assessed potential fixed effects for each model—mass, age, sex, molt status, air temperature and water temperature—for collinearity using scatterplot matrices. Animal location (ASLC, LML) was not included as a fixed effect, as the inclusion of air and water temperature accounted for regional differences. Although photoperiod is known to affect the timing of molt in seals (Mo *et al.*, 2000), we did not include it in our models as photoperiod is strongly correlated with molt status, and our aim was to determine the effect of molt status, not photoperiod, on metabolism.

Prior to model building we assessed dependent variables for normality. Spotted seal absolute $\dot{\textrm{V}}$O_2_ data were log transformed to improve normality. Ringed seal absolute $\dot{\textrm{V}}$O_2_ data were normally distributed and no transformation was needed. For spotted seals and ringed seals, age and mass were highly collinear and age was subsequently dropped. In addition, we examined data for homogeneity of variance. Sex violated the homogeneity of variance assumption using Bartlett’s test for both spotted seals and ringed seals. Given the inclusion of only one female and one male in the study for spotted and ringed seals, respectively, we dropped sex as a fixed factor in both models. We used backwards elimination and corrected Akaike’s Information Criteria (AICc) for model selection. Finally, residuals of the final models were plotted to confirm homoscedasticity and normality, and thus proper model selection.

**Table 2:** Summary of molt and metabolic data for all study animals. Data are presented as means ± standard deviations with sample sizes displayed in parentheses. Molt cycle sample sizes refer to the number of documented molt cycles, while metabolic sample sizes refer to number of data points

Species	Individual	Typical molt timing	Molt duration (d)	Absolute RMR (L O_2_ min^−1^)			Mass-specific RMR (ml O_2_ min^−1^ kg^−1^)		
				Annual mean	Non-molting	Molting	Annual mean	Non-molting	Molting
*Phoca largha*	Amak	May–Jun	38±13 (4)	0.43±0.08 (154)	0.42±0.06 (131)	0.51±0.13 (23)	6.98±1.54 (154)	6.68±1.34 (131)	8.69±1.48 (23)
	Tunu	May–Jun	31±6 (4)	0.36±0.06 (172)	0.35±0.06 (150)	0.42±0.06 (22)	5.47±1.00 (172)	5.34±0.96 (150)	6.41±0.75 (22)
	Sura	Apr–May	36±21 (3)	0.28±0.06 (106)	0.27±0.04 (84)	0.33±0.08 (22)	6.49±1.07 (106)	6.22±1.04 (84)	7.53±1.08 (22)
	Kunik	Apr–May	27±2 (3)	0.25±0.07 (110)	0.24±0.05 (92)	0.31±0.11 (18)	5.58±1.03 (110)	5.35±0.78 (92)	6.79±1.31 (18)
*Pusa hispida*	Nayak	Mar–Apr	36±8 (3)	0.25±0.03 (122)	0.24±0.03 (110)	0.30±0.03 (12)	8.93±1.29 (122)	8.78±1.21 (110)	10.31±1.21 (12)
	Pimniq	May–Jun	21±1 (2)	0.18±0.05 (42)	0.17±0.04 (35)	0.25±0.06 (7)	6.37±1.66 (42)	5.90±1.29 (35)	8.72±1.33 (7)
	Dutch	May	26 (1)	0.16±0.05 (11)	0.13±0.03 (7)	0.21±0.03 (4)	5.73±1.73 (11)	4.68±0.95 (7)	7.57±1.04 (4)
*Erignathus barbatus*	Noatak	Dec–Apr	119±2 (3^*^)	0.48±0.04 (95)	0.47±0.04 (60)	0.50±0.05 (35)	4.10±0.47 (95)	3.96±0.33 (60)	4.34±0.56 (35)

^*^Actual start date of molt could not be determined for this seal during its first year; thus, average molt duration for this animal was determined using two of three molt cycles during the study.

For bearded seal data analysis we began by using multiple linear regressions. Absolute $\dot{\textrm{V}}$O_2_ data were log transformed to improve normality. We assessed potential fixed effects (age, mass, molt status, air temperature and water temperature) for collinearity using scatterplot matrices. Age and mass, as well as air and water temperature were highly correlated. Age and air temperature were subsequently dropped. Using backwards elimination and corrected Akaike’s Information Criteria (AICc) in model selection, we found there was no appropriate regression model to describe the bearded seal data. Therefore, we directly tested the effect of molt status on absolute $\dot{\textrm{V}}$O_2_ using a one-way ANOVA with Tukey’s *post hoc* comparisons. Homogeneity of variance was confirmed using Bartlett’s test.

All statistical analyses were completed using JMP14 statistical software program (SAS Institute, Cary, NC). Metabolic data are presented as mean values ±SD. Results were considered significant if *P* < 0.05.

**Figure 1:**
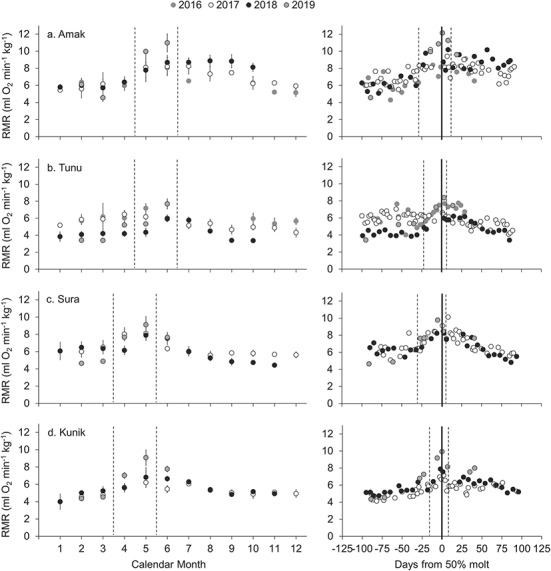
Longitudinal RMR data collected for four spotted seals (a–d) over a 4-year period. Data from each individual in successive years are stacked and displayed in two adjacent panels. Left panels display monthly mean mass-specific RMR (±SD) for each animal. On average, four data points (range: 1–11) were collected each month. Dashed vertical lines denote the typical molting period. Source data for the left panels, along with corresponding absolute RMR values and subject metadata, are provided as Supplementary Data. Right panels display individual metabolic data points collected during the 100 days preceding and 100 days following the 50% molt date each year (solid vertical line). Dashed vertical lines denote the mean start and end dates of molt referenced to the 50% molt date.

**Figure 2:**
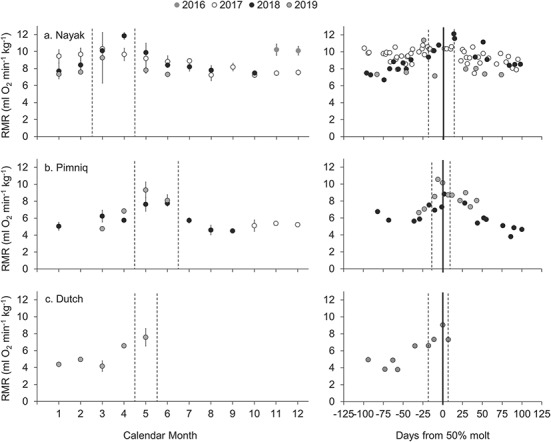
Longitudinal RMR data collected for three ringed seals (a-c) over a four-year period. Data from each individual are stacked and each individual’s data are displayed in two adjacent panels. Left panels display monthly mean mass-specific RMR (±SD) for each animal. On average, three data points (range: 1–10) were collected each month. Dashed vertical lines denote the typical molting period. Source data for the left panels, along with corresponding absolute RMR values and subject metadata, are provided as Supplementary Data. Right panels display individual metabolic data points collected during the 100 days preceding and 100 days following the 50% molt date each year (solid vertical line). Dashed vertical lines denote the mean start and end dates of molt referenced to the 50% molt date.

## Results

Over a four-year period, we collected 812 metabolic data points from eight individuals across three species. Specifically, we collected 542 RMR measurements from four spotted seals, 175 measurements from three ringed seals and 95 measurements from one bearded seal (Table 1). On average, four data points were collected for each individual per month. To account for differences in sample sizes between individuals when determining species-average RMR values we calculated a grand species mean from individual seal means. Although there were intraspecific differences in metabolism, interspecific demands for these individuals followed expected metabolic relationships based on body size (Table 2). Ringed seals had the lowest absolute energy demands (0.20 ± 0.04 L O_2_ min^−1^), but the highest mass-specific demands (7.01 ± 1.38 ml O_2_ min^−1^ kg^−1^). Spotted seals exhibited a mean absolute RMR of 0.33 ± 0.07 L O_2_ min^−1^, with a mean mass-specific RMR of 6.13 ± 0.73 ml O_2_ min^−1^ kg^−1^. The bearded seal had the highest absolute RMR (0.48 ± 0.04 L O_2_ min^−1^) and lowest mass-specific RMR (4.10 ± 0.47 ml O_2_ min^−1^ kg^−1^).

We documented apparent species-level differences in the timing and duration of the annual molt. Spotted and ringed seals molted over relatively short time frames, 33 ± 4 days and 28 ± 6 days, respectively. In contrast, the bearded seal exhibited prolonged molting intervals that lasted an average of 119 ± 2 days. Within individuals, the timing and duration of the annual molt was consistent across years (Table 2), but there was some variation between individuals. Spotted seals generally molted between April and June in Alaska, with the two immature spotted seals beginning their molt a month earlier than the two adults. Both ringed seals in Alaska molted over a similar time frame (May–June), while the ringed seal in California molted earlier; generally, between March and April. The bearded seal molt, which lasted approximately 4 months, consistently occurred between December and April in California.

We observed clear annual patterns in RMR for spotted (Fig. 1) and ringed (Fig. 2) seals that were highly consistent within individuals across years, with both absolute and mass-specific energy expenditure peaking coincident with the annual molt. The longitudinal metabolic patterns for spotted seals were strongly driven by molt status (F_3,534_ = 67.79, *P* < 0.001), air temperature (F_1,533_ = 24.71, *P* < 0.001) and body mass (F_1,482_ = 101.26, *P* < 0.001). RMR increased during molt, as well as with increasing body mass and air temperature (Table 3). Individual accounted for 46% of the observed variance in the spotted seal data. Annual patterns in RMR for ringed seals were driven most strongly by molt status (F_3,168.1_ = 43.91, *P* < 0.001) and water temperature (F_1,170_ = 5.35, *P* = 0.022). For ringed seals, RMR increased in association with the annual molt and decreased with increasing water temperature (Table 4). Individual accounted for 83% of the observed variance for ringed seals. For both species, molt status had a strong effect on metabolism. Mean molting RMR values—in both absolute and mass-specific terms—were consistently higher than non-molting values (Table 2; Supplementary Data), with maximum energetic costs occurring coincident with the 50% new coat date for both species (Figs 1, 2); for some individuals RMR more than doubled at this time relative to non-molting levels.

**Table 3:** LME model best fit results for spotted seals with molt status (F_3,534_ = 67.79, *P* < 0.001), air temperature (F_1,533_ = 24.71, *P* < 0.001) and body mass (F_1,482_ = 101.26, *P* < 0.001) as fixed effects against absolute $\dot{\textrm{V}}$O_2_. Individual was included as a random effect and accounted for 46% of the variance in the model

Parameters	β	S.E.	DF_Den_	t-ratio	P
Intercept	−0.8161	0.044	11.08	−18.44	<0.001
Molt – during	0.0529	0.006	533	8.33	<0.001
Molt – no	−0.0566	0.005	534.1	−12.10	<0.001
Molt – 1 mo. post	0.0225	0.008	535	2.97	0.003
Air Temp	0.0043	0.001	533.3	4.97	<0.001
Mass	0.0053	0.001	481.8	10.06	<0.001

**Table 4:** LME model best fit results for ringed seals with molt status (F_3,168_ = 43.91, *P* < 0.001) and water temperature (F_1,170_ = 5.35, *P* = 0.022) as fixed effects against absolute $\dot{\textrm{V}}$O_2_. Individual was included as a random effect and accounted for 83% of the variance in the model

Parameters	β	S.E.	DF_Den_	t-ratio	P
Intercept	0.2399	0.039	2.42	6.18	0.016
Molt – during	0.0373	0.005	168.1	7.09	<0.001
Molt – no	−0.0349	0.004	168	−9.80	<0.001
Molt – 1 mo. post	0.0051	0.006	168	0.85	0.395
Water Temp	−0.0035	0.001	170	−2.31	0.022

**Figure 3:**
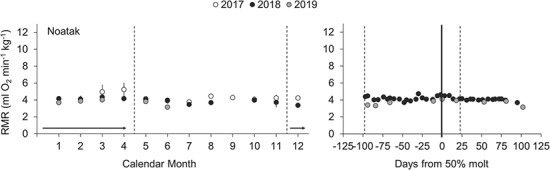
Longitudinal RMR data collected for one bearded seal over 3 years. Left panel displays monthly mean mass-specific RMR (±SD). On average, four data points (range: 1–6) were collected each month. Typical molt period denoted by a horizontal arrow between dashed vertical lines. Source data for the left panel, along with corresponding absolute RMR values and subject metadata, are provided as Supplementary Data. Right panel displays individual metabolic data points collected in the 100 days preceding and 100 days following the 50% molt date each year (solid vertical line). Dashed vertical lines denote the mean start and end dates of molt referenced to the 50% molt date.

In contrast to spotted and ringed seals, the bearded seal did not exhibit notable seasonal patterns or discrete peaks in metabolism during the study period (Fig. 3). Despite comparably stable absolute and mass-specific RMR values (Table 2; Supplementary Data), we did discern an effect of molting on bearded seal metabolism (one-way ANOVA, F_3,91_ = 4.51, *P* = 0.005), with the only significant *post-hoc* comparison between molting and non-molting periods (Tukey’s HSD, *P* = 0.003). However, the percentage difference in mean mass-specific RMR between molting and non-molting periods was much smaller for the bearded seal (9%) than for the spotted seals (18–26%) and ringed seals (16–47%).

## Discussion

### Baseline energy demands

Through the collection of fine-scale longitudinal data we were able to provide year-round, empirical RMR data for eight individuals comprising three Arctic seal species. Energy demands between species followed predicted trends based on body size with the bearded seal exhibiting the highest absolute and lowest mass-specific mean annual RMR, followed by the spotted seals and then the ringed seals. Most notably, our data revealed annual patterns in RMR that were strongly related to the distinct molting strategy of each species. Although we could not establish population-level values due to limited sample sizes for each species, by testing individual seals multiple times per month while in a consistent and calm state, we were able to establish high repeatability for our metabolic measurements both within individuals and across years. Thus, we are confident in the regularity of the annual patterns described here. Further, these types of fine-scale data are valuable for parameterizing predictive population and bioenergetics models.

Given that metabolic data are only available for a limited number of phocid seals, the values reported here improve our understanding of phocid energetics and provide species-specific information pertaining to the metabolic needs of ice-associated seals. Specifically, we contribute metabolic data for the smallest phocid species (i.e*.* ringed seal) as well as the largest Arctic phocid (i.e*.* bearded seal). Indeed, this is the first study to empirically measure metabolism in a bearded seal, which exhibited highly stable annual energy demands. The mean mass-specific RMR we determined for spotted seals was slightly lower than the value previously reported by Ashwell-Erickson et al. (1986); however, only immature individuals (≤2 yrs. old) were measured in that study, which would be expected to have elevated mass-specific demands relative to mature individuals (Kleiber, 1961). Further, as the seals in that study had not been conditioned to participate voluntarily in data collection, they may not have been in a true resting state during metabolic measurements. Thus, our slightly lower values likely reflect the increased age and larger body size of our spotted seals as well as our training protocol. For ringed seals, we found a higher mean RMR in-water than that reported by Ochoa-Acuña et al. (2009) in-air, who collected metabolic data for a single adult male in a haul-out chamber. Of note, the authors provided one in-water value for the same individual in that study, which was nearly twice that of his in-air RMR, but comparable to the mean in-water RMR values reported here.

As would be expected, there were some differences in mean RMR between individuals of the same species. Between-individual differences were minor in spotted seals and pronounced in ringed seals. Across our ringed seals, the greatest differences were observed between the ringed seal in California and those in Alaska. The female ringed seal in California had higher absolute and mass-specific energy demands than either ringed seal in Alaska, likely driven by differences in temperature regime and associated proportions of lean body mass and blubber. We do not believe the ringed seal in California was housed in conditions outside her thermoneutral zone (TNZ), as there was overlap between the ambient temperature ranges at both facilities; however, TNZ has not been established for this species and therefore this possibility cannot be ruled-out. Importantly, although RMR varied between individuals, we observed the same relative patterns across all three ringed seals with similar changes in RMR associated with molt.

### Metabolic consequences of molt

We documented annual changes in RMR that were strongly related to species-specific molting phenology. Spotted and ringed seals molted over relatively short periods and exhibited markedly increased RMRs during this time, which peaked midway through the visible molt. This pattern was considerably different than that of the bearded seal, which took approximately four times longer to molt and exhibited a more subtle increase in RMR across the molting period. This prolonged molting strategy is unique among phocid seals. Although the bearded seal in this study molted earlier than free-ranging conspecifics, the duration and diffuse pattern of molt documented here agree with accounts of wild bearded seals (for review, see Cameron et al., 2010). Further, latitudinal gradients and ontogenetic differences in the timing of molt are common among Arctic seals; seals in lower latitudes tend to molt earlier than seals in higher latitudes likely driven by differences in photoperiod (Mo *et al.*, 2000), and younger seals tend to molt before mature seals (for review, see Boveng et al., 2008; Boveng et al., 2009; Cameron *et al.*, 2010; Kelly et al., 2010). Thus, given that our bearded seal was a young individual (1–3 years) housed in California under local photoperiod, his earlier molting period aligns with geographic and ontogenetic trends in molt timing observed for wild seals. Similarly, the ringed seal housed in California molted earlier than the two ringed seals housed in Alaska. All spotted seals housed in Alaska molted around the same time generally reported for their wild counterparts, with the two younger seals molting prior to the two sexually mature individuals.

The annual molt is generally considered an energetically expensive period for phocids (Boyd *et al.*, 1993; Renouf and Gales, 1994; Boily, 1995, 1996; Boily and Lavigne, 1997; Hedd *et al.*, 1997; Chabot and Stenson, 2002; Williams *et al.*, 2011; Walcott *et al.*, 2020); however, there are inconsistencies in the literature (Ashwell-Erickson *et al.*, 1986; Worthy *et al.*, 1992; Rosen and Renouf, 1998) that have led to the suggestion of a typical pattern of little to no cost of molt in phocids (Beltran *et al.*, 2018). In those species for which we have strong evidence of increased metabolic costs during molt, it is unclear whether the main drivers of those additional costs relate to energetic requirements of generating new hair or to thermal losses associated with sending blood to the periphery to maintain the elevated skin temperatures necessary for cellular growth (Boily, 1995). Our data reveal significant, short-term increases in RMR during the molt for ringed and spotted seals, but also suggest that these costs may be mitigated in some species (i.e*.* bearded seals) by molting over a longer time frame.

The majority of studies examining phocid molting energetics have used methods similar to those described here. Of those studies, significant molt-associated metabolic costs have been reported for grey seals (Boily, 1996; Boily and Lavigne, 1997), harp seals (Hedd *et al.*, 1997; Chabot and Stenson, 2002) and Hawaiian monk seals (Williams *et al.*, 2011); however, metabolic depression during molt has been reported for spotted and harbor seals (Ashwell-Erickson *et al.*, 1986; Rosen and Renouf, 1998). It is not apparent why there would be species-specific differences in the metabolic consequences of molt across phocids; however, resolution of this issue may be found through closer examination of the underlying data in the literature. For example, while Ashwell-Erickson *et al.* (1986) has long been cited as evidence of metabolic depression during molt, the authors actually documented increasing metabolic rates during the regenerative phase of molt. It appears that the interpretation of metabolic depression during molt was based on the early decline in metabolism observed during the 20–60 days preceding the visible molt. When considering only the visible molt, during which time hair is actively shed and regrown, their data largely agree with our findings—and those of other investigators—of additional costs associated with molting.

Increased levels of thyroid hormones have been linked with increased metabolism during the molt. In addition to their effect on metabolism, thyroid hormones stimulate the growth of new fur (Ramot *et al.*, 2009). Elevated thyroid hormones during the visible molt have been documented in grey (Boily, 1996) and harp (John *et al.*, 1987) seals, both species for which there are known metabolic costs associated with molt (Boily and Lavigne, 1997; Hedd *et al.*, 1997; Chabot and Stenson, 2002). Similarly, in penguins, increased thyroid hormone levels are associated with new feather synthesis and increased metabolism (Groscolas and Cherel, 2014). Routti *et al.* (2013) proposed an energetic cost of molt for ringed seals given a documented rise in thyroid hormones during this period, which was confirmed by the direct measures of RMR presented here. In spotted seals, thyroid hormones decline to a minimum just before the molting period and then increase to their maximum values during the period of most rapid hair growth (Ashwell-Erickson *et al.*, 1986). This peak in spotted seal thyroid hormones during the period of most rapid hair growth support the increase in RMR documented in the present study, with peak RMR coinciding with the 50% molt date. Interestingly, in harbor seals—the only other species besides spotted seals for which a metabolic depression during molt has been proposed (Ashwell-Erickson *et al.*, 1986; Rosen and Renouf, 1998)—there are no documented changes in thyroid hormones in association with molting (Renouf and Brotea, 1991).

Although not well studied, it has long been believed that wild bearded seals undergo a protracted molt that differs from other phocids. Some have suggested that the diffuse molt of bearded seals involves year-round shedding of hair with a peak in molting activity during the spring when seals are regularly observed hauling out (for review, see Cameron *et al.*, 2010). Data from the bearded seal in this study supports this notion and a similar molting pattern was observed in two additional captive individuals (C. Reichmuth, unpublished data). Our bearded seal was housed under local photoperiod in California, rather than an Arctic photoperiod, but we do not believe this was the cause of its prolonged molt. Although photoperiod is known to affect timing of molt in seals (Mo *et al.*, 2000), its effect on duration is less clear. Indeed, the ringed seal housed in California consistently had an earlier onset of molt than the ringed seals in Alaska, but the progression and duration of molting was similar for all ringed seals. Thus, photoperiod was likely responsible for the early onset of the bearded seal’s molt, but not a direct cause of its prolonged duration.

The pronounced elevation in RMR we observed in spotted and ringed seals during molt was noticeably absent in the bearded seal, with no observable increase associated with the 50% molt date. By having a diffuse molt over an extended period, bearded seals may limit the overall energetic cost of this annual event and/or spread out the costs to reduce the metabolic impact at any one point in time. This energy-saving strategy may be facilitated by the much larger body size of bearded seals relative to other ice seals. This strategy may be physiologically impractical for ringed and spotted seals due their smaller body sizes, higher surface area to volume ratios and potential rates of heat loss, which may necessitate more rapid molt cycles. However, there are likely other trade-offs associated with this protracted molting strategy that should be considered, such as the impact on haul-out behavior. Overall, our results support the notion of an extended molt in bearded seals, enhance our understanding of individual molt dynamics and reveal the potential benefits of this unique strategy.

One conflicting finding from our study was the species-specific effects of air and water temperature on metabolism. RMR increased with increasing temperature in spotted seals, but decreased with increasing temperature in ringed seals. Further, we found no effect of temperature on the bearded seal’s RMR. While additional thermoregulatory costs can be accrued when animals are in environments outside their TNZ, this seems unlikely here. Based on studies of harbor seals, ambient temperatures experienced by our spotted seals were likely within their TNZ (Hansen, 1995; Hansen *et al.*, 1995). The pattern we observed for ringed seals might be explained if individuals were tested below their lower critical temperature at points during the study. However, the lowest water temperature experienced by our ringed seals was 4.0 °C, a temperature well within the normal range of their wild counterparts (Kelly *et al.*, 2010). Therefore, rather than temperature driving observed patterns in RMR, it is more likely that the timing of molt is associated with seasonal changes in temperature that do not themselves have independent effects on metabolism.

### Conservation implications

Climate change is a pervasive threat to all ice-associated seals (Kovacs *et al.*, 2011; Laidre *et al.*, 2015), although the ability of these seals to adjust to changing conditions will depend on the unique characteristics of each species. Here, we provide some of the most comprehensive metabolic data for spotted, ringed and bearded seals with which to better predict the species-specific consequences of ongoing Arctic warming. These data fill important gaps in our understanding of the physiology of each species, and provide source data for predictive population and bioenergetic models that are important for conservation and management efforts. Further, this work highlights species-specific physiological attributes that are important when considering the impact of sea ice loss during key life-history events, such as molt.

In polar regions, the increased skin temperatures that are necessary to promote tissue regeneration during molt can only be achieved by ice seals when hauled out (Feltz and Fay, 1966; Boily, 1995; Walcott *et al.*, 2020). If seals were unable to do so, increased skin perfusion in polar water for prolonged periods would result in untenably high rates of heat loss (Watts *et al.*, 1993). Thus, adequate haul-out substrate is essential for polar seals during molt, raising specific concerns about the physiological consequences of declining sea ice. We found that spotted and ringed seals exhibit significant peaks in energy expenditure associated with their discrete molt and we observed them spending more time hauled out during this period than at any other time during the year. Despite the fact that our bearded seal exhibited minimal changes in metabolism associated with molt, we also observed his haul-out time to increase markedly during molt, particularly from around the 50% molt date through the end of molt. This suggests benefits of increased skin temperatures for molting bearded seals, despite their muted metabolic response.

Declining ice cover can also place energetic burdens on ice-dependent seals in other ways. To ensure adequate haul-out substrate during molt, there is evidence that ringed seals remain with preferred sea ice habitat as it retreats, even into marginal foraging areas (Hamilton *et al.*, 2015; Von Duyke *et al.*, 2020). Our data suggest that this behavior reflects the bioenergetic priorities of seals during molt. The advantages of hauling out to promote tissue regeneration may outweigh the caloric benefits of remaining in preferred foraging areas. This behavioral change occurs despite the additional energetic investment that individuals must make to move with or travel to preferred sea ice regions. Bearded seals tend to select areas of broken, drifting pack ice over shallow foraging grounds (Burns, 1967; Smith and Stirling, 1975; Bengtson *et al.*, 2005). As this type of sea ice retreats from preferred foraging grounds, suitable haul-out substrate for bearded seals may become decoupled from important regions. This would force bearded seals to either move to areas of preferred ice cover, as has been observed for ringed seals, or to move coastal regions to haul out on land where risk of predation will be greater (Smith, 1980). Tagging data from juvenile bearded seals suggests they respond similarly to ringed seals, opting to move with preferred sea ice as it retreats (Breed *et al.*, 2018; Cameron *et al.*, 2018).

Although behavioral adjustments may currently allow seals to maintain contact with preferred sea ice habitat during molt, continued sea ice loss will likely require individuals to spend greater amounts of time in water. It remains unclear whether individuals can successfully molt without hauling out (Feltz and Fay, 1966; Boily, 1995). If seals curtail surface blood flow in response to spending more time in water, or cannot maintain elevated skin temperatures, this could slow, delay, or disrupt the overall process of tissue regeneration. Recent unusual mortality events (UMEs) for ice associated pinnipeds (2011–2016; 2018–present) may provide glimpses into the physiological consequences of reduced sea ice during molt (NOAA Fisheries, 2011a, 2011b, 2016, 2020). Although the cause of the 2011–2016 Alaska Pinniped UME is still unknown, seals and walruses presented with abnormal skin lesions and what appeared to be abnormal or disrupted molts (NOAA Fisheries, 2018). This UME coincided with many years of early sea ice breakup. A more recent UME, specific to ringed, bearded and spotted seals, began in 2018 and is ongoing (NOAA Fisheries, 2020). As part of this UME, seals are presenting with similar symptoms and appear in poor body condition, suggesting widespread energetic losses. These UMEs may be some of the first examples of the detrimental effects of the loss of sea ice for ice-associated seals.

The 2012 decision to list distinct population segments of bearded seals as threatened under the United States Endangered Species Act was driven by concern that sea ice loss would substantially reduce or eliminate adequate haul-out platforms for pupping and molting, leading to decreases in reproduction and survival (NMFS, 2012). Similar concerns exist for ringed seal populations, which are predicted to be at risk of major declines due to ongoing sea ice loss (Kelly *et al.*, 2010; Kovacs *et al.*, 2011; Reimer *et al.*, 2019). Spotted seals may be less impacted by reductions in sea ice as they are already known to successfully utilize coastal haul outs for pupping and molting in lower latitudes (Wang, 1986; Nesterenko and Katin, 2009); however, the cascading consequences of climate change will inevitably affect all three species. Ultimately, this work highlights potential energetic trade-offs associated with different molting strategies in these three species and more broadly, provides valuable quantitative data regarding annual patterns in energy demands that can be used to assess species-specific vulnerabilities of Arctic seals to changing conditions.

## Funding

This work was supported by the National Oceanic and Atmospheric Administration’s Alaska Pinnipeds Program [NA15NMF4390166, NA16NMF4390027, NA19NMF4390083].

## Supplementary Material

Supplimentary_Data_coaa112
